# ER Stress-Induced Secretion of Proteins and Their Extracellular Functions in the Heart

**DOI:** 10.3390/cells9092066

**Published:** 2020-09-10

**Authors:** Bianca A. Meyer, Shirin Doroudgar

**Affiliations:** 1 Department of Internal Medicine III (Cardiology, Angiology, and Pneumology), Heidelberg University Hospital, Im Neuenheimer Feld 669, 69120 Heidelberg, Germany; biancaannameyer@t-online.de; 2 DZHK (German Centre for Cardiovascular Research), Partner Site Heidelberg/Mannheim, 69120 Heidelberg, Germany

**Keywords:** protein secretion, ER stress, unfolded protein response (UPR), proteostasis, secreted ER chaperones, cell signaling, cardiokines, cardiac myocytes

## Abstract

Endoplasmic reticulum (ER) stress is a result of conditions that imbalance protein homeostasis or proteostasis at the ER, for example ischemia, and is a common event in various human pathologies, including the diseased heart. Cardiac integrity and function depend on the active secretion of mature proteins from a variety of cell types in the heart, a process that requires an intact ER environment for efficient protein folding and trafficking to the secretory pathway. As a consequence of ER stress, most protein secretion by the ER secretory pathway is decreased. Strikingly, there is a select group of proteins that are secreted in greater quantities during ER stress. ER stress resulting from the dysregulation of ER Ca^2+^ levels, for instance, stimulates the secretion of Ca^2+^-binding ER chaperones, especially GRP78, GRP94, calreticulin, and mesencephalic astrocyte-derived neurotrophic factor (MANF), which play a multitude of roles outside the cell, strongly depending on the cell type and tissue. Here we review current insights in ER stress-induced secretion of proteins, particularly from the heart, and highlight the extracellular functions of these proteins, ranging from the augmentation of cardiac cell viability to the modulation of pro- and anti-apoptotic, oncogenic, and immune-stimulatory cell signaling, cell invasion, extracellular proteostasis, and more. Many of the roles of ER stress-induced protein secretion remain to be explored in the heart. This article is part of a special issue entitled “The Role of Proteostasis Derailment in Cardiac Diseases.”

## 1. Protein Secretion in the Heart

**Figure 1:**
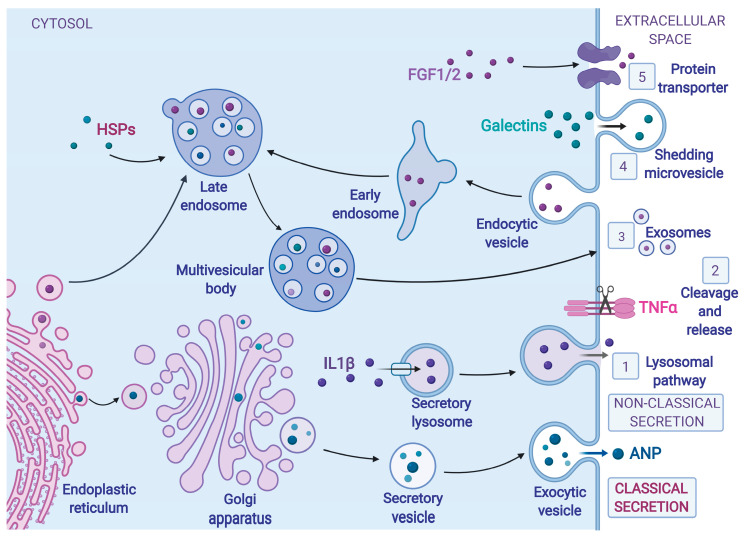
Mechanisms of Cardiokine Secretion. Cardiokines (secreted proteins from cardiac cells), such as atrial natriuretic protein (ANP), made at the endoplasmic reticulum (ER), are conventionally transported to the Golgi apparatus, where they are modified and packaged into secretory vesicles and then to the plasma membrane, where they are released into the extracellular space, in what is known as classical protein secretion. Some cardiokines that enter the ER bypass the Golgi apparatus, in some cases entering membrane-bound organelles such as endosomes, on their way to the plasma membrane. Cardiokines secreted via the non-classical secretory pathway are synthesized on cytosolic ribosomes and traffic through various kinds of either vesicle- or nonvesicle-mediated pathways, without transiting through the ER and the Golgi apparatus. Non-classical protein secretion pathways include (1) incorporation into the lysosomal pathway, for example, interleukin 1β (IL1β), (2) cleavage and release from a membrane-bound precursor, for example, tumor necrosis factor α (TNFα), (3) release via exosomes derived from multivesicular bodies, for example, heat shock proteins (HSPs), (4) membrane blebbing and microvesicle shedding, for example, galectins, and (5) direct translocation of proteins across the plasma membrane through membrane transporters, for example fibroblast growth factor 1 and 2. Figures were created with BioRender.com.

The heart is made of highly differentiated cells that must communicate with each other and with cells outside the heart to facilitate normal organ development and function [1]. The four most abundant cardiac cell types are fibroblasts, endothelial cells, cardiac myocytes, and smooth muscle cells [2]. Active protein secretion is a crucial element of intercellular communication and mediates autocrine, paracrine, or endocrine signaling, depending on whether secreted proteins have an effect on the secreting cell, neighboring cells, or distant tissues, respectively. Passive secretion, by contrast, refers to the release of cellular contents from dying cells, following necrotic tissue damage. Proteins actively secreted from cardiac cells are called cardiokines [3,4] and are released via the classical [5] or non-classical [6] secretory pathways (Figure 1). In the classical pathway, proteins are secreted after they are synthesized and translocated into the endoplasmic reticulum (ER). They are then transported to the Golgi and subsequently secreted after secretory vesicles fuse with the plasma membrane. Non-classical protein secretion does not involve the ER-Golgi pathway.

In classical protein secretion, the mRNAs encoding many secreted and membrane proteins are specifically localized to the cytosolic face of the ER membrane by several mechanisms, the most studied of which involves signal recognition particles (SRP) [7]. In eukaryotes, SRP binds to the signal sequence of a newly synthesized peptide as it emerges from the ribosome and through interactions with the SRP receptor, targets the ribosome-nascent chain complex to dock with the protein-conducting channel, also known as the translocon, in the ER membrane. Upon ribosome docking to the ER, nascent polypeptides are co-translationally inserted into the ER lumen the translocon [8]. SRP-independent pathways for the delivery of mRNAs to the ER have been proposed [9,10], with new evidence suggesting that in addition to SRP-targeted signal sequences in the peptide nascent chain, alternative signal motifs in the mRNA may also be responsible for directing protein synthesis to the ER [11]. Subsequent to protein folding and modification, proteins are routed in vesicle-mediated transport alongside the ER-Golgi intermediate compartment (ERGIC) and the Golgi apparatus towards their final destination [12]. The last step of classical protein secretion can be constitutive or regulated, depending on whether the secretory vesicles fuse with the plasma membrane directly after packaging, or whether they are stored until the appropriate stimulus activates their secretion (Figure 1; Classical secretion). One prominent example of a classically secreted cardiokine is the atrial natriuretic peptide (ANP), or atrial natriuretic factor (ANF), a small peptide hormone that reduces blood pressure and volume in an endocrine manner; ANF is released from atrial myocytes by regulated secretion in response to atrial stretch [13,14,15]. 

Non-classical secretion, on the other hand, describes ER-/Golgi-independent protein trafficking through various kinds of either vesicle- or nonvesicle-mediated pathways [16]. At least five non-classical protein secretion pathways have been described [17]. They include: (1) lysosomal secretion, (2) cleavage and release of a membrane-bound protein, (3) release via exosomes derived from multivesicular bodies, (4) membrane blebbing, and (5) direct translocation of proteins across the plasma membrane through membrane transporters (Figure 1; Non-classical secretion 1–5). One important example, S100A1, a cardiac myocyte-specific member of the EF-hand Ca^2+^-binding S100 protein family, is a regulator of cardiac myocyte function and is secreted via the non-classical secretory pathway during myocardial infarction. Extracellular S100A1 is taken up by cells via Ca^2+^-dependent, clathrin-mediated endocytosis and protects cardiac myocytes from apoptosis by signaling through phospholipase C (PLC) and protein kinase C (PKC) associated with endosomes [18].

Taken together, actively secreted proteins play many roles in the healthy heart, as well as in the damaged heart, including cardiac tissue remodeling and repair [3]. Considering the role of the ER in classical protein secretion, the status of the ER is an important determinant of cardiac function.

## 2. ER Stress in the Heart

### 2.1. Cardiac Proteostasis at the ER

The quality and quantity of cellular protein is managed by an extensive network of molecular mechanisms that collectively constitute the dynamic balance of protein homeostasis, or proteostasis [19]. Proteostasis involves the synthesis, folding, and trafficking of proteins, as well as the degradation of excess protein, terminally unfolded, or misfolded proteins, and responses to stresses that risk protein quality by dysregulating proteostasis. Proteostasis is essential for cellular function in and outside the heart [20], leading to longer life span when maintained properly [21]. Accordingly, cardiac ageing is associated with gradual derailment of cardiac proteostasis, which underlies the development of numerous cardiac diseases [22]. An optimal ER environment is required for efficient protein synthesis, folding, and modification. Many proteins need to be folded into the appropriate three-dimensional configuration to be functional [23]. Efficient protein folding in the ER is determined by the availability of ER Ca^2+^ [24], *N*-linked glycosylation [25], redox status of the ER with regards to disulfide bond formation [26], and the abundance of ER-resident molecular chaperones that assist in protein folding [27]. Terminally unfolded or misfolded proteins, which have failed dynamic protein folding and quality control, are removed from the ER via the ubiquitin/proteasome-dependent process of ER-associated protein degradation (ERAD) [28]. Since there are no proteasomes in the lumen of the ER, misfolded proteins in the ER must be translocated out of the ER and into the cytosol, where they are ubiquitylated by ER-transmembrane E3 ubiquitin-protein ligases and subsequently degraded by proteasomes on the cytosolic face of the ER [29]. In addition to the ER, specialized cells types, such as cardiac myocytes, comprise further intracellular membrane networks, i.e., the sarcoplasmic reticulum (SR). The attachment of ribosomes to the SR suggests that protein synthesis may also take place at the SR [30]. The SR is best known for its function in Ca^2+^ storage, release, and reuptake for excitation-contraction coupling in muscle cells [31]. Nonetheless, proper definition and distinction of the ER and SR with regards to essential components of cellular proteostasis, for example localization of protein synthesis, remain to be determined.

### 2.2. ER Stress and Heart Disease

**Figure 2:**
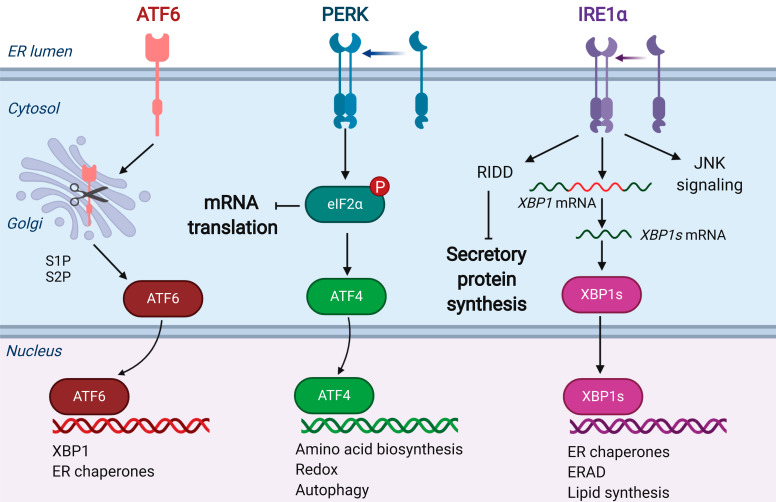
Regulation of ER Stress by the Unfolded Protein Response and Effects on Cardiokine Secretion. Perturbations of the ER environment result in the activation of the unfolded protein response, mediated by ER-resident integral membrane proteins ATF6 (activating transcription factor 6), PERK (protein kinase RNA (PKR)-like ER kinase), and IRE1α (inositol requiring enzyme 1). Upon ER stress, ATF6 translocates to the Golgi apparatus, where it is cleaved by site-1 and site-2 proteases (S1P, S2P), liberating an N-terminal fragment to translocate to the nucleus, where it is a potent and labile transcription factor that activates the transcription of ER chaperones and the transcription factor X-box binding protein 1 (XBP1). Activation of ATF6 may increase ER secretory capacity [35]. PERK dimerization activates autophosphorylation of its kinase domain, leading to phosphorylation of the α-subunit of translation initiation factor 2 (eIF2α) at Ser51. This inhibits global translational initiation, including translation of mRNAs encoding secretory proteins, reducing the load of unfolded proteins entering the ER. PERK activation also results in the induction of translation of selective mRNAs, including activating transcription factor 4 (ATF4), which activates the transcription of a wide range of genes involved in adaptation to stress conditions. Upon activation, IRE1α oligomerizes and carries out regulated IRE1-dependent decay (RIDD) of ER-targeted transcripts, as well as unconventional RNA splicing, by removing an intron from the XBP1 mRNA, allowing it to become translated into the functional transcription factor, XBP1s. XBP1s activates the transcription of ER chaperones, endoplasmic reticulum associated degradation (ERAD) genes, and genes involved in lipid synthesis.

Age- and disease-linked alterations of the ER environment, or genetically encoded mutations that misbalance proteostasis at the ER, lead to a mismatch of ER protein folding capacity and load, resulting in ER stress and the accumulation of misfolded proteins in the ER [32]. Misfolded proteins not only lack biological function, but may gain potentially toxic activity by exposing hydrophobic regions to nearby proteins, which can result in inappropriate binding and aggregation of nonfunctional structures [33]. To avoid proteotoxic effects in stressed cells, the ER senses protein folding status within the ER by monitoring levels of folding-deficient proteins, which leads to the activation of a conserved signaling network, called the unfolded protein response (UPR) [34] (Figure 2).

As a consequence, ER protein folding capacity is enhanced to match its demand by focusing cellular resources on synthesizing proteins that are involved in the folding process, such as protein disulfide isomerases and ER-resident chaperones [36]. Meanwhile, ER protein folding load is reduced by suppression of overall cellular protein synthesis [36], along with the augmentation of ER-associated degradation (ERAD) of misfolded proteins [37]. Together, these aspects of the UPR ultimately contribute to relieve ER stress [38]. Three ER-resident integral membrane proteins, PERK (protein kinase RNA (PKR)-like ER kinase) [39,40], IRE1α (inositol requiring enzyme 1α) [41,42,43,44], and ATF6 (activating transcription factor 6) [45,46,47], function as ER stress sensors (Figure 2). Downstream UPR signaling can be activated by the dissociation of the ER-luminal chaperone, glucose-regulated protein 78 kDa (GRP78), from the ER-transmembrane sensors, to assist the refolding of misfolded proteins or direct them towards ERAD [48]. Additional evidence suggests that misfolded proteins can also directly associate with the ER stress sensors, particularly IRE1α [49]; however, the exact mechanisms by which ER stress is sensed are still not clearly understood. Upon activation, PERK, IRE1α, and ATF6 transmit the information about detected ER stress into the cytosol through regulation of translational control (PERK), mRNA splicing (IRE1α), and regulated proteolysis (ATF6). From there, the corresponding transcription factors ATF4, XBP1, and ATF6 enter the nucleus, where they bind to specific elements in ER stress response genes and regulate their transcription [34]. Overall, the UPR is oriented toward the reestablishment of proteostasis in order to promote cell survival, which is adaptive. However, if the adaptive UPR is insufficient to reestablish proteostasis, depending on the strength and duration of ER stress, the UPR becomes maladaptive, guiding the cell towards apoptosis [50,51,52]. In particular, prolonged activation of the PERK branch of the UPR determines the apoptotic cell fate [53]. Programmed cell death upon unresolved ER stress is an especially important problem in the heart, given the limited regenerative potential of cardiac muscle [54]. 

UPR signaling is activated in cardiac myocytes under various pathologic conditions, such as ischemia, hypertrophy, or heart failure [55,56,57]. A prominent pathologic setting that causes ER stress in the heart is ischemia, the deprivation of oxygen and nutrient delivery to cardiac cells due to inadequate blood flow, often as a result of atherosclerosis or myocardial infarction. While ischemic cardiac myocytes in an infarct zone undergo cell death within 15–30 min of loss of blood supply, the surviving myocardium in the periphery of an infarct is exposed to milder ischemia [58]. Several studies have aimed to determine the extent to which each UPR pathway and subcellular signaling of its components are adaptive and can be used for therapeutic approaches. For example, under conditions when energy sources are not abundant, such as ischemia, activation of the ATF6 branch of the UPR reprograms cardiac myocytes in ways that limit growth, thus, conserving energy [56,59]. In this case, the induction of anti-growth-oriented genes during ER stress is considered protective, since it contributes to protect cardiac tissue against pathological hypertrophy, which is a hallmark of the diseased heart [60]. However, the UPR is multifaceted and under other conditions, when energy sources are abundant, ATF6 serves as regulator of the induction of major growth-promoting pathways in cardiac myocytes [61].

## 3. Protein Secretion during ER Stress

Cardiac proteostasis is crucial for the secretion of mature, properly folded proteins from cardiac cells. ER stress impairs most of protein processing and transport through the classical secretory pathway, ultimately limiting active protein secretion in stressed cells [62]. In addition to PERK-dependent translational attenuation, the UPR contributes to the reduction of ER protein trafficking by regulated IRE1α-dependent decay of mRNA (RIDD) [63], a mechanism of selective degradation of ER-bound mRNAs, and selective release of secreted and membrane protein-encoding mRNAs from the ER to the cytosol [64]. Secretion of selective proteins, however, is induced during ER stress, even when secretion of most other proteins that are secreted via the ER-Golgi secretory pathway has been attenuated. Proteins selectively secreted during ER stress fall into at least one of two categories: (1) functional proteins that are secreted upon the pathologic alteration of the ER environment by a distinct stress stimulus, i.e., decreased ER Ca^2+^ levels during ischemia [65], and (2) functional proteins whose secretion is directly regulated by the UPR [66]. Both kinds of proteins can exert protective effects after secretion, from outside cells, which will be discussed in the following sections in more detail.

## 4. Secretion of Ca^2+^-Binding ER Chaperones

### 4.1. Secretion during Ca^2+^-Mediated ER Stress

Ca^2+^ handling in cardiac cells is essential for cardiac contractibility [67]. Dysregulation of Ca^2+^ handling in cardiac myocytes contributes substantially to the initiation and progression of cardiac arrhythmia and heart failure [68]. ER Ca^2+^ depletion by chemical stressors, for example thapsigargin, a strong inhibitor of ER Ca^2+^-ATPases, is a common method for examining the effects of ER stress, in particular ER stress mediated by ER Ca^2+^ depletion [69]. Thapsigargin mimics the pathological decrease of ER Ca^2+^ levels during ischemic conditions, under which, for example, ATP production and thereby the activity of ATPases is limited. Moreover, the majority of ER-resident molecular chaperones and folding enzymes (protein disulfide isomerases and peptidyl prolyl isomerases) are low-affinity, high-capacity Ca^2+^-binding proteins and the activities of many of these are Ca^2+^-regulated [70,71]. Therefore, chaperone and folding functions are disturbed upon perturbation of ER-luminal Ca^2+^ levels [72,73,74,75].

Studies in which ER stress is induced either by ER Ca^2+^ depletion, for example by thapsigargin treatment or, alternatively, by ER protein glycosylation inhibition, by compounds such as tunicamycin, have shown that only Ca^2+^-mediated ER stress induces the secretion of a select group of ER proteins [62,65]. These findings suggest that different ER stressors have different effects on protein secretion and highlight the particular importance of altered ER Ca^2+^ levels for ER stress-induced protein secretion. Several studies in cardiac myocytes, fibroblasts, and other cell types, have reported on the secretion of ER-luminal chaperones with Ca^2+^-binding properties during Ca^2+^-mediated ER stress. Among these proteins are GRP78, GRP94, calreticulin [65], and mesencephalic astrocyte-derived neurotrophic factor (MANF) [76], all of which require Ca^2+^ for essential protein-protein interactions. In the ER, these proteins are typically involved in protein, as well as, Ca^2+^ homeostasis. Beyond the ER, they can be found, in part, in various other subcellular compartments, including the cytoplasm, nucleus, mitochondria, and the cell surface, with distinct functions depending on their localization [77,78,79] and novel functions continuously being discovered [80].

ER-luminal protein mobility was first observed in the 1990s in immunogold electron microscopy studies that showed, for example, the export of GRP78 and GRP94 from the ER and routing through the classical secretory pathway in rat exocrine pancreatic cells [81]. Although these Ca^2+^-binding ER chaperones possess a C-terminal retention motif that facilitates binding to the KDEL-receptor [82], which fosters the retrieval of proteins from the *cis*-Golgi back to the ER [83], they manage to overcome ER retention under certain conditions, such as ER Ca^2+^ depletion. Current explanations include an overwhelmed capacity of the KDEL retrieval system, alteration of KDEL system components, masking of the KDEL motif, for example by glycosylation of the protein sequence adjacent to KDEL [84], and decreased ER retention caused by drastically reduced ER Ca^2+^ levels during certain ER stress [85]. Notably, GRP78, GRP94, calreticulin, and MANF are also ER stress-inducible transcriptional targets of the UPR, particularly of the transcription factors ATF6 and XBP1. Nevertheless, their secretion happens within minutes of ER Ca^2+^ depletion, suggesting that it is independent from their transcriptional induction [76]. Since their transcripts efficiently escape translational inhibition, UPR-induced upregulation of these proteins can increase their extracellular levels further.

### 4.2. Extracellular Functions of ER Chaperones in the Cardiovascular System

**Figure 3:**
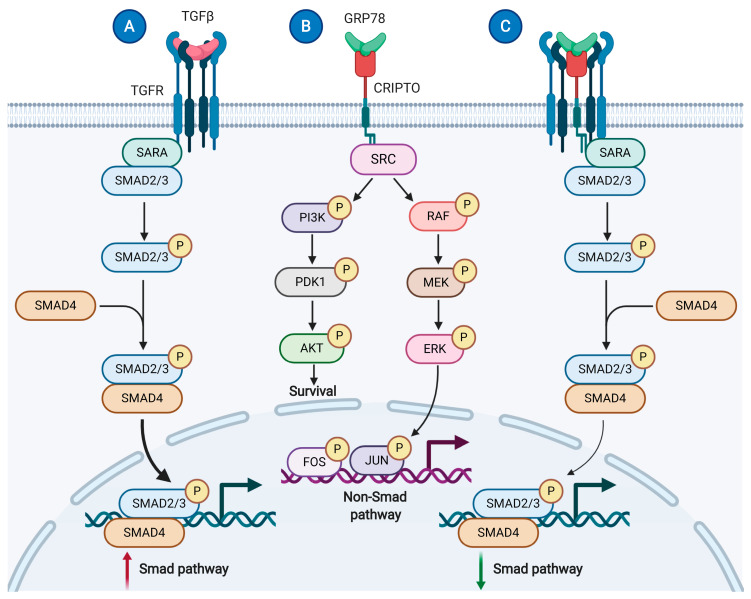
Model of extracellular GRP78 and interacting protein, Cripto, modulation of TGFβ signaling. (**A**) Transforming growth factor beta (TGFβ) ligand binding to TGFβ receptors (TGFR) leads to activation of genes responsive to Smad2/3 signaling. (**B**) The 78 kDa glucose regulated protein (GRP78), an ER luminal Ca^2+^-binding chaperone, is secreted upon ER Ca^2+^ depletion. Secreted GRP78 is a co-factor required for Cripto-1 growth factor (Cripto) activation. Cripto activates Src, Ras/Raf/MAPK and PI3K/Akt pathways via a mechanism that remains unknown. (**C**) Cell surface Cripto/GRP78 interaction is required for Cripto modulation of TGFβ signaling.

Accumulating evidence suggests that secreted Ca^2+^-binding ER chaperones in the heart contribute to enhanced cardiac cell viability and cardioprotection. Our recent study on the cardiac myocyte secretome during ER stress reported the selective secretion of ER-resident proteins, i.e., GRP78, calreticulin, and GRP94, from cardiac myocytes in vitro upon ER Ca^2+^ depletion by thapsigargin or stimulated ischemia [62]. These proteins were cytoprotective in a culture media volume-dependent, auto- and paracrine manner, supporting their potential role in the regulation of cell viability in the onset of Ca^2+^-mediated ER stress. Mechanistically, GRP78 was shown to interact with the protein, Cripto, on the cell surface of cardiac myocytes to activate pro-survival AKT signaling and to inhibit death-promoting SMAD2 signaling (Figure 3A–C). This study demonstrated for the first time that GRP78 secreted as a result of ER stress can protect cardiac myocytes in a Cripto-dependent manner. The small, glycosylphosphatidylinositol (GPI)-anchored signaling protein, Cripto, plays important roles in vertebrate embryogenesis as well as tumorigenesis (e.g., inhibition of TGFβ signaling) [86,87]. Previously, GRP78 was identified as a necessary mediator of oncogenic Cripto signaling via MAPK/PI3K and Smad2/3 pathways at the cell surface of non-cardiac cell types, such as cancer cells [88,89]. Importantly, in addition to its secretion in response to ER stress, a subfraction of GRP78 can relocate to the cell surface during ER stress [84], where it resides mostly as a peripheral protein via interaction with other proteins [90], and acts as a multifunctional receptor in various signaling pathways [91] (see more in Section 4.3). Moreover, Bi et al. showed that overexpression of GRP78 in neonatal rat ventricular myocytes protected myocytes from ischemia/reperfusion-induced cell death, and cardiac myocyte-specific overexpression of GRP78 decreased ischemia/reperfusion damage to the heart [92]. GRP78-mediated cytoprotection involved plasma membrane translocation of GRP78 and interaction with PI3 kinase, resulting in AKT activation.

Another study identified GRP78 on the surface of endothelium and monocyte/macrophage-like cells in atherosclerotic lesions, where it was shown to negatively regulate tissue factor (TF) procoagulant activity via direct binding to and functional inhibition of TF [93]. Meanwhile, calreticulin protects against mild ischemia/reperfusion injury via association with the cardiokine C1q/TNF-related protein 9 (CTRP9) on the cell surface of cardiac myocytes through activation of the PKA/CREB pathway, ultimately inhibiting cardiac myocyte apoptosis [94]. Moreover, MANF is also known to be selectively released during Ca^2+^-mediated ER stress, including hypoxia, ischemia, and treatment with thapsigargin, and after its release, MANF protects cardiac myocytes from ischemia/reperfusion damage in vitro and in vivo [76,85]. MANF family proteins are highly evolutionarily conserved and both human and Drosophila orthologues promote dopamine neuron survival [95,96,97,98]. For over a decade since the cytoprotective effects of extracellular MANF were first reported [95,99], no receptor for MANF was known. However, recently, lipid sulfatide, also called 3-O-sulfogalactosylceramide, a sulfoglycolipid synthesized in ER and Golgi, which distributes to the extracellular leaflet of the plasma membrane of many eukaryotic cells, was reported to act as a direct mediator of MANF’s cytoprotective function, as it is located in the outer cell membrane of target cells and promotes cellular uptake of MANF by endocytosis [100]. Overall, our knowledge of the adaptive roles for ER stress-induced Ca^2+^-binding chaperone secretion from cardiac cells is finite, yet very promising. Therefore, identifying and understanding the mechanisms of action of these kinds of secreted proteins remains a key challenge of the field.

### 4.3. Extracellular Functions of ER Chaperones in Non-Cardiovascular Cells and Tissues

Secreted and cell surface-associated GRP78, GRP94, calreticulin, and MANF have been investigated in many non-cardiac cell types in different contexts, including ER stress. To complement the available knowledge of extracellular functions of these proteins in the heart, we have decided to include in this review findings pertaining to the extracellular functions of these proteins from other fields. This, we hope, can help guide future studies of these proteins in the heart. Therefore, this section aims to provide a short overview of extracellular functions identified for these proteins outside the heart.

#### 4.3.1. 78 kDa Glucose-Regulated Protein (GRP78; Binding Immunoglobulin Protein)

In recent years, the critical role of cell surface and secreted GRP78 in the tumor microenvironment, and its potential as a target in anti-cancer therapies, have become well-recognized [101]. For example, GRP78 is preferentially present on the surface of cancer cells, where it collaborates with other surface proteins to mediate anti-apoptotic and proliferative signaling [102]. Furthermore, GRP78 secreted from tumor cells was recently shown to act as a chemokine that recruits macrophages into tumor tissues through cytoskeletal remodeling [103]. Another study reported the translocation of GRP78 to the cell surface and a novel role of GRP78 in neuroprotection, as it contributes to a decrease in maladaptive PERK activation at the onset of ER stress by direct binding to serine protease tissue-type plasminogen activator (tPA) at the cell surface [104]. In pancreatic beta cells, by contrast, inflammatory cytokines induce surface translocation of GRP78, where it initiates pro-apoptotic signaling cascades, including increased caspase 3/7 activity, as well as Chop and Bax mRNA expression. This study found that GRP78 is shuttled through the Golgi apparatus and secretory granules, and identified the DNAJ homolog subfamily C member 3 (DNAJC3) as a GRP78-interacting protein that facilitates its membrane translocation. The pro-apoptotic signaling was found to be mediated by extracellular, soluble GRP78 binding to cell surface GRP78 [105]. In different cell types, extracellular GRP78 displays anti-inflammatory and immunomodulatory properties [106]; for example, GRP78 can mediate endocytosis of toll-like receptor (TLR) 4 with CD14 to resolve inflammation [107]. At the cell surface, GRP78 can also act as a co-receptor to modulate cell invasion, i.e., virus entry in host cells, as shown, for example, for Coxsackie virus A9 [108], dengue virus serotype 2 [109], Borna disease virus [110], and Japanese encephalitis virus [111]. Previously, GRP78 was reported to augment virus attachment of certain coronavirus lineages (e.g., MERS-CoV) onto host cells [112], to which a recent study added the prediction of GRP78 binding sites to the spike proteins of the novel SARS-CoV-2, the strain of coronavirus that causes coronavirus disease 2019 (COVID-19) [113]. This prediction was made based on molecular modeling by docking and structural bioinformatics, and is highly relevant in consideration of the current worldwide COVID-19 pandemic.

#### 4.3.2. Other Ca^2+^-Binding ER Chaperones: Calreticulin, 94 kDa Glucose-Regulated Protein (GRP94; Endoplasmin; Gp96), MANF

Since the identification of calreticulin in the extracellular matrix [114], its secretion, and localization to the cell surface, particularly upon reduced ER Ca^2+^ [65,115], studies have reported extracellular functions of calreticulin in multiple physiological and pathological processes. These include cutaneous wound healing [116], immunological responses, i.e., induction of immunogenic cancer cell death [117] and promotion of phagocytic uptake of cancer [118], viable and apoptotic cells [119] as an “eat me” signal, fibrosis [120], cancer cell survival [121], and more. Previous reviews have highlighted the multifunctionality of calreticulin outside the ER [24,78], which is underscored by the continuous characterization of its extracellular protein interactions and functions [122]. By contrast, extracellular GRP94 has been acknowledged, thus far, mostly for its contribution to immunological responses, e.g., by GRP94 surface binding, uptake, and peptide cross-presentation [123], and delivery of co-secreted client proteins [124]. Additionally, proinflammatory properties of GRP94 were elucidated in GRP94 overexpressing transgenic mice, where overexpression of an engineered form of GRP94 that remains on the cell surface induced dendritic cell activation and spontaneous autoimmune diseases [125]. Originally identified as a secreted trophic factor in neurons, protective roles of secreted MANF continue to be discovered in various contexts, including neuroprotection [97], cardioprotection (see, Section 4.2.), and diabetes (reviewed in [126]).

## 5. Secretion of Other Proteins during ER Stress

### 5.1. Other Cardiokines

Ischemia in vivo and simulated ischemia in vitro, the latter of which is defined as 0.1% O_2_ and lack of glucose, cause ER stress in cardiac cells [3,56]. A recent study of the secretome of primary cardiac myocytes provided evidence that protein secretion can be regulated by hypoxia [127]. The secreted proteins were found to contribute to the cellular response to hypoxia, the immune system, cell death, and cellular wounding. Moreover, the study showed increased secretion of the calcium-dependent serine endoprotease proprotein convertase subtilisin/kexin type 6 (PCSK6), an unexplored protein in cardiac ischemia, from cardiac myocytes by hypoxia. Significant increases in PCSK6 mRNA and protein levels were detected in hypoxic cardiac myocytes, infarct and infarct border zone, as well as in blood samples from patients suffering from acute ST-elevation myocardial infarction. Secreted PCSK6 was found to regulate TGFβ signaling and fibrosis in cardiac fibroblasts. Regulation of expression and secretion of PCSK6 during ER stress conditions, including ischemia in glucose-free conditions and lower oxygen levels, remain to be elucidated.

While some cardioprotective secreted proteins, such as follistatin-like 1 (Fstl1) and tumor necrosis factor (TNF)-α, can still be secreted during ischemic stress, secretion of other cardiokines, for example, encephalin and calcitonin gene-related peptide, is even enhanced by mild ischemia, which contributes to preserving cardiac function [3]. For an overview of ischemic stress-induced protein secretion in the heart the readers are referred to [3].

### 5.2. ERdj3 (Endoplasmic Reticulum DNA J Domain-Containing Protein 3; DnaJ Homolog Subfamily B Member 11)

ERdj3, a chaperone of the heat shock protein (HSP)-40 family, is an exemplar of how intracellular and extracellular proteostasis are linked, particularly during ER stress. In the ER, ERdj3 normally directs misfolded proteins to GRP78 [128]. In contrast to GRP78, ERdj3 lacks a KDEL ER retention motif. Therefore, at least half of newly synthesized ERdj3 is secreted via the classical secretory pathway. In 2015, Genereux et al. demonstrated that ERdj3 is an UPR-induced secreted chaperone, whose extracellular levels increase in response to ER stress and stress-independent activation of the UPR-associated transcription factor ATF6 [66]. Increased secretion of ERdj3 acts in extracellular proteostasis in two ways: (a) Free ERdj3 binds and attenuates extracellular misfolded protein aggregates that escaped ER protein quality control. (b) ERdj3 is co-secreted with destabilized, presumably misfolded protein clients when the chaperone capacity of intracellular GRP78 has been overwhelmed. Both mechanisms protect tissues from the potentially toxic extracellular aggregation of proteins, a maladaptive issue that contributes to the pathological phenotypes of many protein misfolding diseases. It also suggests a shift of proteins involved in extracellular proteostasis during ER stress. Secretion of the prominent extracellular chaperone, clusterin, for example, is reduced under similar ER stress conditions, in a process involving stress-induced retrotanslocation out of the ER and into the cytosol [129].

### 5.3. Angiogenin

Angiogenin, a cytoplasmic ribonuclease, is a secreted RNase of the pancreatic RNase A superfamily, showing tRNA substrate cleavage, and consequently inhibiting protein synthesis [130,131]. Mutations in angiogenin are linked to the development of amyotrophic lateral sclerosis (ALS) [132]. Recombinant angiogenin extends the lifespan of transgenic ALS mice [133] and protects primary motoneurons against hypoxic injury [134]. Angiogenin is secreted from neurons, and then endocytosed by astroglia; in this way, angiogenin mediates neuroprotection in a paracrine manner by eliciting astrocytes to induce RNA cleavage [135]. During pathologic conditions, for instance in acute kidney injury, cytoplasmic angiogenin interferes with translational initiation through cleavage, and thereby production of tRNA fragments, which contributes to decreased protein synthesis and alleviation of ER stress [136]. In a recent study on UPR signaling in the kidney, novel extracellular functions of angiogenin have been identified [137]. This study found that during ER stress, angiogenin expression and secretion are increased. Angiogenin secretion is under the selective control of ER stress sensor and UPR regulator, IRE-1*α*. The transcription factors, XBP1s and p65, which are activated by IRE-1*α* upon ER stress, bind to the angiogenin promoter, thereby upregulating angiogenin expression, leading to increased secretion. Similar to secretion of the ER stress-induced proinflammatory cytokine IL-6, secretion of angiogenin requires the ER-Golgi pathway. Upon secretion, extracellular angiogenin promotes macrophage reprogramming towards an activated and proinflammatory phenotype through receptor-mediated PI3K/AKT signaling and/or activation of intracellular pattern recognition receptors by tRNA fragments produced by angiogenin after endocytosis. Therefore, angiogenin has been characterized as a mediator of an ER stress-dependent inflammatory response of acute kidney ischemia that promotes kidney tissue adaption [137]. It remains to be determined whether ER stress-induced secretion of ribonucleases plays a role in tissues other than the kidney.

## 6. Conclusions and Future Directions

Characterization of proteins whose secretion is activated, maintained, or enhanced during ER stress holds promising therapeutic potential for a variety of human diseases, including heart diseases, as well as protein misfolding and autoimmune diseases, and cancer. Our view of ER chaperones has evolved considerably over the past two decades and affirmed their immense multifunctionality through ever-expanding findings of their roles inside and outside cells. Moreover, the UPR has been redefined as a critical regulator of cellular proteostasis that has the potential to link intra- and extracellular mechanisms of stress management. Considering the importance of maintaining precise control over intracellular Ca^2+^ levels that regulate cardiac myocyte contraction, further studies of Ca^2+^-mediated ER stress-inducible protein secretion could contribute substantially to our understanding of how secreted proteins can mediated cardioprotection.

In summary, most ER chaperones secreted during ER stress are secreted due to decreased ER Ca^2+^. Moreover, ERdj3 is the first metazoan chaperone whose secretion is directly regulated by the UPR. Considering that ER proteostasis involves a number of chaperones whose novel functions are continuously being discovered, the importance and innovation capacity of this field is highlighted here. We expect further characterization of the underlying mechanisms of secretion and extracellular actions of these kinds of proteins in the future, by examining their roles in pro-survival, oncogenic, and immune-stimulatory cell signaling, extracellular proteostasis, and more, both inside and outside the heart.
